# Mechanical Stretch Suppresses microRNA-145 Expression by Activating Extracellular Signal-Regulated Kinase 1/2 and Upregulating Angiotensin-Converting Enzyme to Alter Vascular Smooth Muscle Cell Phenotype

**DOI:** 10.1371/journal.pone.0096338

**Published:** 2014-05-21

**Authors:** Bo Hu, Jian tao Song, Hai yan Qu, Chen long Bi, Xiao zhen Huang, Xin xin Liu, Mei Zhang

**Affiliations:** Key Laboratory of Cardiovascular Remodeling and Function Research, Chinese Ministry of Education and Chinese Ministry of Health, Department of Cardiology, Qilu Hospital, Shandong University, Jinan, Shandong, People’s Republic of China; Federal University of São Paulo (UNIFESP), Escola Paulista de Medicina, Brazil

## Abstract

Phenotype modulation of vascular smooth muscle cells (VSMCs) plays an important role in the pathogenesis of various vascular diseases, including hypertension and atherosclerosis. Several microRNAs (miRNAs) were found involved in regulating the VSMC phenotype with platelet-derived growth factor (PDGF) treatment, but the role of miRNAs in the mechanical stretch-altered VSMC phenotype is not clear. Here, we identified miR-145 as a major miRNA contributing to stretch-altered VSMC phenotype by miRNA array, quantitative RT-PCR and gain- and loss-of-function methods. Our data demonstrated that 16% stretch suppressed miR-145 expression, with reduced expression of contractile markers of VSMCs cultured on collagenI; overexpression of miR-145 could partially recover the expression in stretched cells. Serum response factor (SRF), myocardin, and Kruppel-like factor 4 (KLF4) are major regulators of the VSMC phenotype. The effect of stretch on myocardin and KLF4 protein expression was altered by miR-145 mimics, but SRF expression was not affected. In addition, stretch-activated extracellular signal-regulated kinase 1/2 (ERK1/2) and up-regulated angiotensin-converting enzyme (ACE) were confirmed to be responsible for the inhibition of miR-145 expression. Mechanical stretch inhibits miR-145 expression by activating the ERK1/2 signaling pathway and promoting ACE expression, thus modulating the VSMC phenotype.

## Introduction

Deregulation of vascular smooth muscle cells (VSMCs) functions plays a critical role in the pathogenesis of many proliferative vascular diseases, including hypertension and atherosclerosis. Unlike some terminally differentiated cells, VSMCs maintain remarkable phenotypic plasticity. Within mature blood vessels, VSMCs rarely proliferate and migrate, they mainly perform contraction function and express a variety of SMC-specific contractile markers, including α-smooth muscle actin (SMA), SM22α and calponin (CNN). However, in response to injury, VSMCs undergo phenotypic modulation, a process characterized by decreased contractile marker expression and increased proliferation, migrate, extracellular matrix (ECM) synthesis. [1], [2]. Altered VSMCs phenotype is essential for vascular development and repair, but deregulation of this process also plays a critical role in pathological vascular remodeling [3]. Thus, a complete understanding of the mechanisms of VSMCs phenotypic modulation will help reveal novel therapeutic targets in vascular diseases.

VSMCs phenotype modulation could occur at different layers of gene expression. Recently, microRNAs (miRNAs) were reported to be involved in the regulation of the VSMC phenotype. MiRNAs are a class of endogenous, small non-coding RNAs that negatively regulate gene expression by directly degrading mRNA or inhibiting translation [4]–[6]. MiRNAs expression is abundant in vascular walls, they almost participate in all cellular functions of vascular cells [7].

Blood vessels are constantly exposed to mechanical forces in the form of shear stress and stretch due to blood flow and blood pressure. Recently, several studies revealed that the miRNA expression profile in cultured endothelial cells (ECs) was significantly altered by shear stress, and miR-21, -19a, -23b, -92a were involved in shear stress-mediated apoptosis, cell cycle, nitric oxide production and inflammation [8]–[11]. In our previous study, we found that miR-21 expression in cultured human aortic smooth muscle cells (HASMCs) was altered by cyclic stretch, which is implicated in proliferation and apoptosis [12]. However, the roles of miRNAs in stretch mediated other cellular functions in cultured SMCs are not clear.

VSMCs are the main target cells of stretch, but the effect of stretch on VSMC phenotype is not consistent in vitro studies [13], [14]. The origin of VSMCs, magnitude of stretch and the ECM used in experiments may contribute to the discrepancy. Several in vitro and in vivo studies demonstrated that miRNAs, especially miR-143/145, were critical regulators of the VSMC phenotype, however, the roles of miRNAs in stretch-induced VSMC phenotype modulation are not clear.

In the present study, we used miRNA array to observe the expression of miRNAs altered by stretch in cultured HASMCs and investigated the regulatory mechanism of miRNAs in the stretch-altered VSMC phenotype.

## Materials and Methods

### Cell Culture and Stretch Application

HASMCs were obtained from ScienCell and cultured in smooth muscle cell medium(SMCM) (USA) with 5% CO_2_ at 37°C. Cells at passages 4–7 were used for experiments. HASMCs were seeded onto Flexcell 6-well plates coated with collagenI, when they reached 80∼90% confluence, serum-free SMCM was replaced to induce quiescence for 24 hr, then cells were exposed to cyclic stretch (16% elongation, 1 Hz) generated by a computer-controlled Flexcell 5000-tension apparatus for the indicated times. The apparatus was kept in a humidified incubator with 5% CO2 at 37°C. Cells cultured in static conditions were static controls. For inhibition of signaling pathway activation, specific Pharmacological inhibitors were added to SMCM 1 hr before stretch treatment.

### miRNA Array

After being stretched for 12 hr or maintained under static conditions, total RNA was harvested using TRIzol (Invitrogen) and miRNeasy mini kit (QIAGEN) according to manufacturer’s instructions. After having passed RNA quantity measurement using the NanoDrop 1000, the samples were labeled using the miRCURY Hy3/Hy5 Power labeling kit and hybridized on the miRCURY LNA Array (v.16.0). Following the washing steps the slides were scanned using the Axon GenePix 4000B microarray scanner. Scanned images were then imported into GenePix Pro 6.0 software (Axon) for grid alignment and data extraction, following with data analysis.

### Quantitive Real-time PCR (qRT-PCR)

For detecting miRNA expression, after being stretched for the indicated time, total RNA, including miRNAs, the level of mature mir-21, -221, -145 was determined by qRT-PCR using the universal cDNA synthesis and SYBR Green Master Mix kits. (Denmark), rRNA U6 expression was used as an internal reference. The miR-specific LNA PCR primer set and the primer for U6 were also obtained from Exiqon.

For detecting pri-miR-143/145 and the mRNA level of angiotensin-converting enzyme (ACE), SM22α, SMA, CNN, total RNA was harvested and reverse transcribed to cDNA using the PrimeScript RT reagent kit (Japan), and real-time PCR involved use of the SYRB Premix Ex Taq kit (Japan). PCR cycling conditions were 95°C for 3 min, 40 cycles of 95°C for 10 sec, 56°C for 10 sec, and 72°C for 10 sec. The primer sequences were as follow: pri-miR-134/145, forward, 5′-AACTCCAGCTGGTCCTTAG-3′, and reverse, 5′-TCTTGAACCCTCATCCTGT-3′; ACE, forward, 5′-GCGGCTCTTCCAGGAGCTGC-3′ and reverse, 5′-CTGCGCCCACATGTTCCCCA-3′; SMA, Forward: 5′-GCGTGGCTATTCCTTCGTTA-3′ and reverse, 5′-ATGAAGGATGGCTGGAACAG-3′; SM22α, Forward: 5′-AACAGCCTGTACCCTGATGG-3′ and reverse, 5′-CGGTAGTGCCCATCATTCTT-3′; CNN, Forward: 5′-AGCTAAGAGAAGGGCGGAAC-3′ and reverse, 5′-CATCTGCAGGCTGACATTGA-3′; GAPDH, forward, 5′-AAGGTGAAGGTCGGAGTC-3′ and reverse, 5′-GATTTTGGAGGGATCTCG-3′. GAPDH was as a internal reference for target mRNAs level.

### Western Blot Analysis

Protein from HASMCs subjected to stretch or not was extracted by use of a cytoplasmic extraction reagent kit (USA). The protein concentrations were determined by the BCA method. Western blot analysis was performed to detect the protein level. In brief, 15–20 µg protein extract was separated by SDS-PAGE and transferred to PVDF membrane (USA), which was blocked with 5% nonfat milk for 2 hr. Then membranes were incubated with primary antibodies overnight at 4°C. After a washing with 1×TBST 3 times, membranes were incubated with secondary antibodies for 1 hr. Visualization involved an enhanced chemiluminescence-plus detection system. Relative band intensities were analyzed by use of Photoshop CS3. The major primary antibodies used were as follow: anti-ACE antibody (abcam, ab28611), anti-CNN antibody (epitomics, #1806-1), anti-SMA(abcam, ab15734), anti-SM22α (abcam, ab14106), anti-SRF (Cell signaling technology, CST, #5147), anti-Kruppel-like factor (KLF4) (CST, #4038), anti-Myocardin (abcam, ab22073), total and phospho-MAPK antibody (CST, #9926, #9910).

### Transient Transfection

Chemically modified miRNA inhibitors or mimics specific for miR-145, -21 and -221 were obtained from GenePharm (Shanghai). The sequence for miR-145 inhibitor was: 5′-AGGGAUUCCUGGGAAAACUGGAC-3′; sequence for miR-145 mimics were: 5′- GUCCAGUUUUCCCGGAAUCCCU-3′and5′- GGAUUCCUGGGAAAACUGGACU-3′; the sequence for miR-21 inhibitor was: 5′-UCAACAUCAGUCUGAUAAGCUA-3′; the sequence for miR-221 inhibitor was: 5′-UCGAUGUAACAGACGACCCAAAG-3′; the sequence for miR-221 mimics were: 5′-AGCUACAUUGUCUGCUGGGCCCU-3′ and 5′-AGAUGCCUGGGAGAACUGGACUU-3′; the sequence for negative control (NC) mimics were: 5′-UUCUCCGAACGUGUCACGUTT -3′ and 5′-AGGUGACACGUUCGGAGAATT -3′; the sequence for the negative control (NC) miRNA inhibitor was: 5′-CAGUACUUUUGUGUAGUACAA-3′. The above miRNAs mimics or inhibitors were transfected into cultured HASMCs using lipo 2000 (USA) at a final concentration of 90 nM before stretch treatment. At 48 hr, HASMCs were exposed to 16% stretch or maintained under static conditions. For down-regulation ACE and KLF4, specific small interfering RNA (siRNA) of the two genes were transfected into HASMCs respectively, by use of Lipo 2000 at a final concentration of 100 nM 48 hr before stretch treatment. The sequence for ACE siRNA were: 5′-CUA UCA AGC GGA UCA UAA A dTdT-3′ and 5′-UUU AUG AUC CGC UUG AUA G dTdT-3′; the sequence for KLF4 siRNA were:

5′-ACCUUGCCUUACACAUGAATT-3′and 5′-GGACCUAGACUUUAUCCUUTT-3′; the sequence for NC siRNA were: 5′-UUCUCCGAACGUGUCACGUTT-3′ and 5′-ACGUGACACGUUCGGAGAATT-3′.

### Measurement of Angiotensin II (AngII)

HASMCs with and without ACE siRNA treatment were exposed to 16% stretch or kept under static conditions for 12 hr, then cytoplasmic protein was extracted and the protein concentration was measured by the BCA method, and the concentration of AngII in the HASMCs lysate was determined by use of an commercially available enzyme-linked immunosorbent assay (ELISA) kit (France).

### Statistical Analysis

All data are shown as mean ± SEM. Statistical significance was determined by one-way ANOVA or two-tailed unpaired Student’s *t* test. *P*<0.05 was considered statistically significant.

## Results

### Differential Expression of miRNAs in HASMCs in Response to Mechanical Stretch

**Figure 1:**
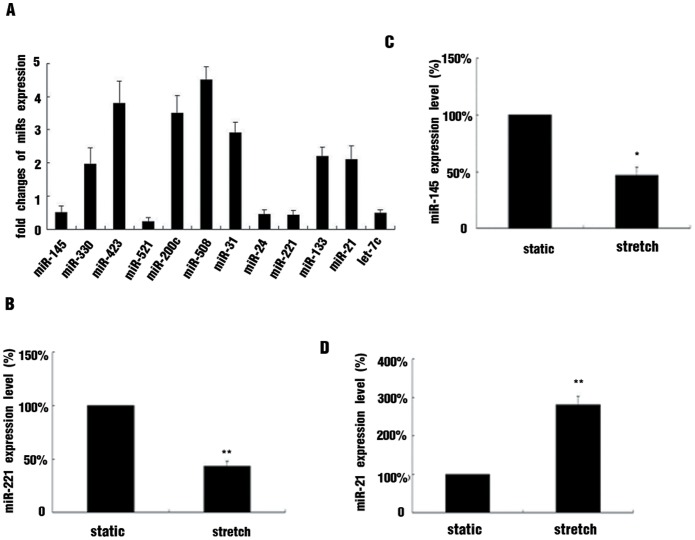
Effect of stretch on miRNA expression profile in cultured human aortic smooth muscle cells (HASMCs). (A) HASMCs were exposed to mechanical stretch (16% elongation, 1 Hz) or maintained under static conditions for 12 hr. MiRarray assay was performed to investigate the stretch-altered expression of miRNAs. P<0.05, vs static, n = 3. (B, C, D) Cultured HASMCs were subjected to 16% stretch for 12 hr or maintained under static conditions, Quantitative RT-PCR (qRT-PCR) analysis of mRNA expression of miR-221, miR-145 and miR-21 under stretch. Data are mean±SEM of 4 experiments. *p<0.05, **p<0.01, vs static.

By use of miR microarray approach, we compared the miRNA profiles in cultured HASMCs subjected to 16% stretch for 12 hr with those maintained under static conditions. Among the miRNAs represented on the microarrays, several were found to be significantly up-regulated or down-regulated (>2-fold) in stretched cells in comparison with the static control cells (Fig. 1A), including miR-145 (0.5-fold), miR-31 (2.9-fold), miR-24 (0.45-fold), miR-133b (2.2-fold), miR-221 (0.43-fold) and miR-21 (2.1-fold). The above mentioned miRNAs were reported to be associated with VSMC phenotype modulation under different conditions. To further verify the effects of stretch on the expression of these miRNAs, we used quantitative real-time PCR (qRT-PCR) to detect their expression; the expression of miR-221, miR-21 and miR-145 in stretched cells was consistent with the microarray results (Fig. 1B, C, D).

### MiR-145 was Involved in Stretch-altered VSMC Phenotype

**Figure 2:**
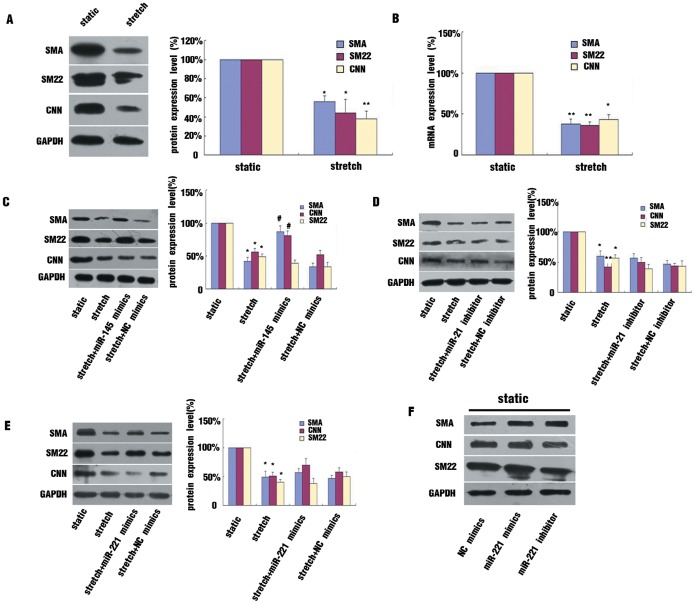
MiR-145 was involved in stretch-altered HASMC phenotype. (A and B) Effect of stretch on expression of SMC contractile markers. HASMCs were exposed to 16% stretch for 12 hr or maintained under static conditions. Western blot and qRT-PCR analysis of protein and mRNA levels of SMC contractile markers, respectively. Normalization was to level of GAPDH. (C, D, E) Modulation of miR-145, -21, -221 levels by use of miRNA mimics or inhibitors in HASMCs exposed to 16% stretch for 12 hr. Western blot analysis and quantification of protein levels of SMC contractile markers. (F) Modulation of miR-221 level in HASMCs under static conditions. Western blot analysis of protein levels of SMC contractile markers. Data are mean±SEM of 4 experiments. *p<0.05, **p<0.01, vs static; # p<0.05, vs stretch.

Stretch affects the cellular functions of cultured VSMCs, including proliferation, apoptosis and differentiation [15]. We previously revealed that 16% stretch increased the proliferation and apoptosis of cultured HASMCs [12]. In the present study, after 12 hr of 16% stretch treatment, the protein and mRNA levels of VSMC contractile markers decreased significantly compared of static controls (Fig. 2A and B). To further verify the miRNAs involved in this process, we used gain- and loss-of-function approaches to evaluate the effects of altered miRNAs on VSMCs phenotype. Our results showed that overexpression of miR-145 by mimics significantly attenuated the stretch-induced inhibition of contractile marker expression in cultured HASMCs (Fig. 2C). However, inhibition of stretch-induced miR-21 expression by a specific miRNA inhibitor had no effect on VSMC contractile markers expression in stretched cells (Fig. 2D), and overexpression of miR-221 in stretched cells also had no effect on VSMC contractile markers expression(Fig. 2E).

MiR-221 is associated with increased VSMC proliferation and is a negative regulator of VSMC contractile phenotype [16]. We used chemically modified miR-221 mimics or inhibitor to regulate its expression in HASMCs maintained under static conditions. The expression of VSMCs contractile markers was not affected by miR-221 mimic or inhibitor(Fig.2F), therefore, miR-145 was implicated in the stretch-decreased expression of VSMCs contractile markers.

### KLF4 and Myocardin were Targets of miR-145 in Stretched Cells

**Figure 3:**
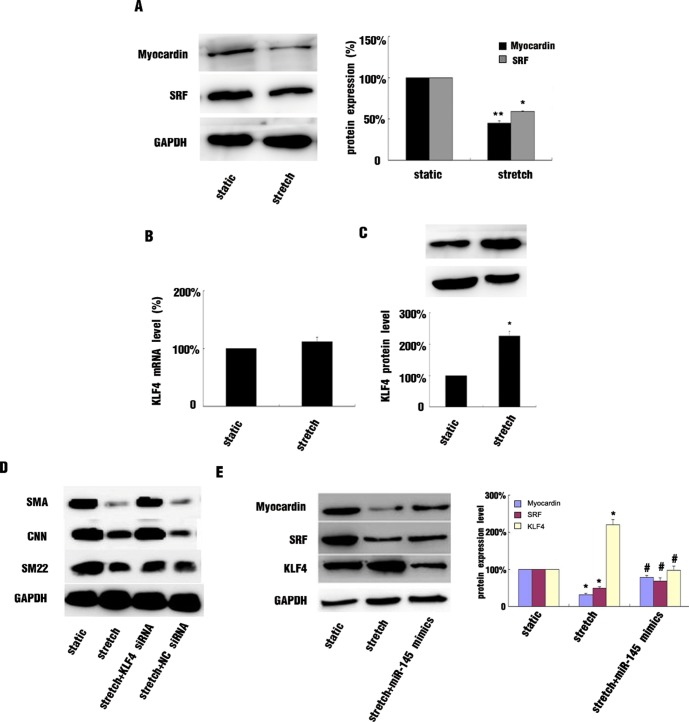
Myocardin and Kruppel-like factor 4 (KLF4) expression was partially modulated by miR-145 in stretched HASMCs. (A) Western blot analysis and quantification of protein levels of myocardin and SRF in HASMCs subjected to 16% stretch or maintained under static conditions for 12 hr. (B and C) Effect of 16% stretch on KLF4 protein and mRNA levels, respectively, in cultured HASMCs. (D) Western blot analysis of protein level of VSMC contractile markers with KLF4 siRNA knockdown and negative control (NC) siRNA knockdown under stretch treatment and (E) Western blot analysis and quantification of protein levels of myocardin, SRF and KLF4 protein level treated with miR-145 mimics in stretched HASMCs. Data are mean±SEM of 4 experiments. *p<0.05, **p<0.01, vs static.

SRF and myocardin are critical positive regulators of VSMC differentiation, and both of them promote the expression of miR-145 in VSMCs, myocardin was also identified as a target of miR-145 [17]. In our study, 16% stretch decreased the protein levels of SRF and myocardin (Fig. 3A). overexpression of miR-145 by miR-145 mimics significantly attenuated stretch-reduced myocardin expression but had no effect on SRF expression in stretched cells (Fig. 3E). KFL4 is also an important target of miR-145, negatively regulating VSMC differentiation. Here, the mRNA but not protein level of KLF4 was increased with stretch treatment (Fig. 3B and C), and the miR-145 mimics almost abolished the stretch-induced protein level of KLF4 (Fig. 3E). We used KLF4 siRNA to suppress its protein level in stretched HASMCs, which increased the expression of VSMC contractile markers as compared with negative control siRNA (Fig. 3D).

### Activation of the ERK1/2 Signaling Pathway Contributed to Stretch-induced Inhibition of miR-145 Expression

**Figure 4:**
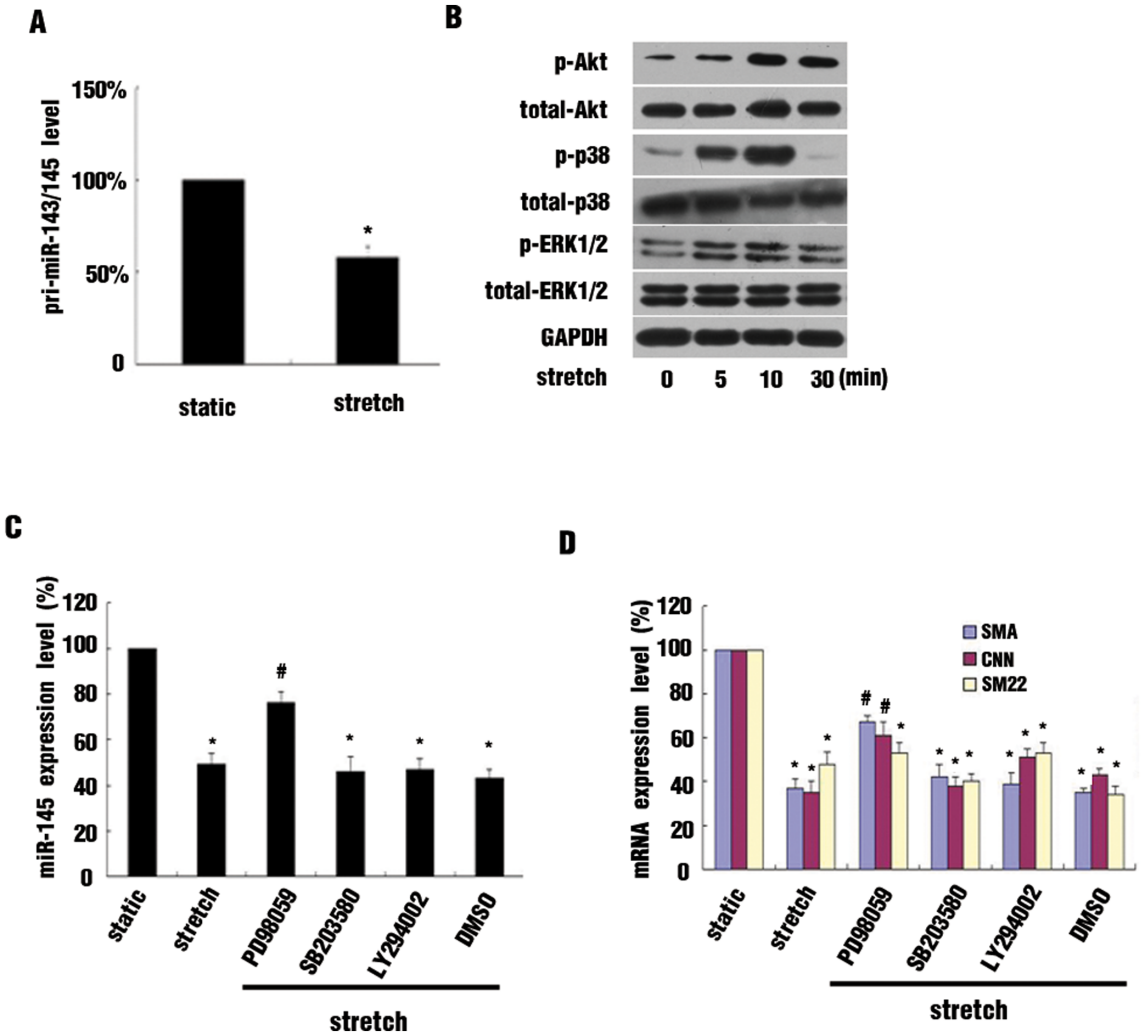
ERK1/2 signaling pathways was involved in stretch-induced miR-145 suppression. (A) Effect of stretch on primary miR-145 (pri-miR-145) expression. qRT-PCR analysis of mRNA level of pri-miR-145 in HASMCs exposed to 16% stretch for 12 hr or maintained under static conditions. (B) Effect of 16% stretch on activation of ERK1/2, p38 MAPK and Akt signaling pathways. Western blot analysis of the activation of signaling pathways. (C and D) ERK1/2 inhibitor PD98059, p38 inhibitor SB203580, Akt inhibitor LY294002 were added to the medium 1 hr before 16% stretch. qRT-PCR analysis of the SMC contractile markers mRNA and miR-145 level in HASMCs. Data are mean±SEM of 4 experiments. *p<0.05, vs static, # p<0.05, vs stretch.

Regulation of miR-145 could occur at transcriptional or post-transcriptional levels, so we investigated the primary miR-143/145 (pri-miR-143/145) level in HASMCs with or without stretch treatment. The pri-miR-143/145 level was reduced in stretched cells as compared with static controls (Fig. 4A), which indicates that stretch-inhibited miR-145 occurred at least in part at the transcriptional level.

Mechanical stretch can rapidly activate several signaling pathways to modulate gene expression. ERK1/2, p38 mitogen-activated protein kinase (MAPK), and Akt signaling pathways are involved in an altered phenotype of VSMCs under various conditions. In our study, these pathways were also rapidly activated, as demonstrated by increased p-ERK1/2, p-p38 MAPK, and p-Akt levels in stretched cells (Fig. 4B). To verify which pathway was responsible for stretch-reduced miR-145 expression, we used the pharmacological inhibitors PD98058, SB23058 and LY294002 to block these signaling pathways. Only the ERK1/2 inhibitor PD98058 significantly attenuated stretch-reduced miR-145 expression (Fig, 4C), which was accompanied by increased expression of VSMC contractile markers (Fig. 4D).

### Up-regulation of ACE Implicated in miR-145 Expression Mediated by Stretch in HASMCs

**Figure 5:**
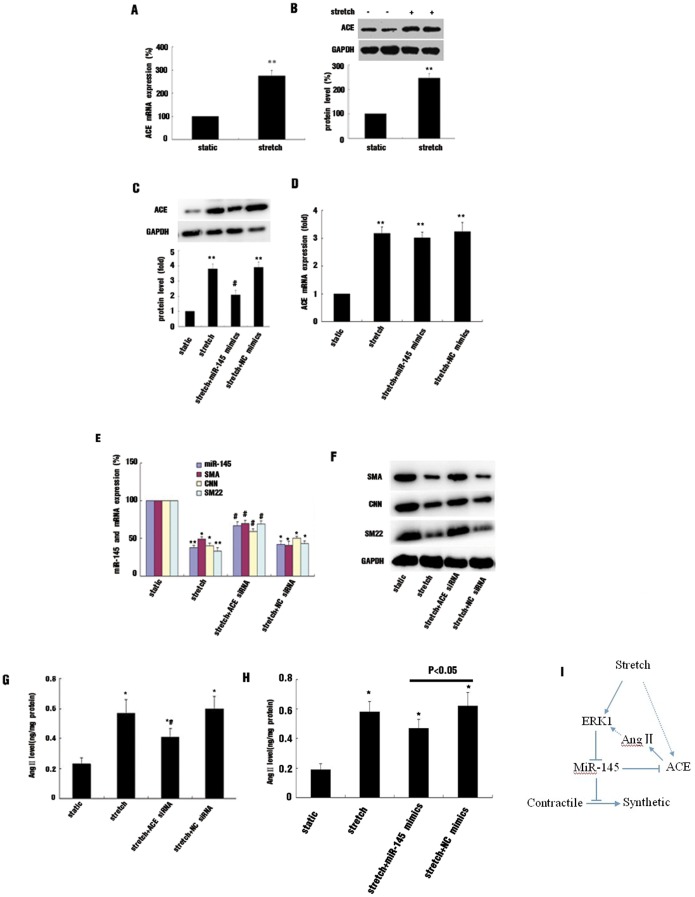
Angiotensin-converting enzyme (ACE) was involved in stretch-induced miR-145 inhibition. (A and B) Effect of stretch on ACE expression. qRT-PCR and western blot analysis of mRNA and protein levels, respectively. (C and D) Effect of miR-145 mimics on protein and mRNA levels of ACE in stretched HASMCs. (E and F) Effect of ACE or NC siRNA on miR-145 level and mRNA and protein levels of VSMC contractile markers in HASMCs, respectively, *p<0.05, **p<0.01, vs static, # p<0.05 vs stretch, n = 4. (G) Effect of ACE siRNA on Angiotensin II (AngII) level in stretched HASMCs. Cells transcribed with ACE or NC siRNA were exposed to 16% stretch for 12 hr. AngII level in cytoplasm was measured by use of ELISA kit; (H) Effect of miR-145 mimics on AngII level in stretched HASMCs; Data are present as mean±SEM of 5 experiments. *p<0.05 vs static, #p<0.05 vs stretch. (I) schematic representation of stretch mediated miR-145 expression and interaction with ACE.

ACE plays a crucial role in catalyzing AngI to AngII, which promotes a VSMC phenotype switch from a contractile to synthetic state. Previous reports found the expression and activity of ACE influenced by shear stress, but the effect of stretch on its expression is unclear. As well, ACE was identified as a target of miR-145 [6]. Our results indicate that 16% stretch significantly enhanced ACE protein and mRNA expression compared to static controls (Fig. 5A and B); overexpression of miR-145 partially attenuated stretch-induced ACE protein expression but had no effect on mRNA level (Fig. 5C and D), so stretch-induced ACE expression at least partially occurred at post-transcriptional level. Using siRNA to knockdown ACE expression attenuated the inhibition of miR-145 expression by 16% stretch, and the expression of VSMC contractile markers in stretched cells was also increased (Fig. 5E and F). AngII level was higher in stretched cells than static controls, and ACE siRNA significantly reduced AngII level in stretched cells compared to control siRNA (Fig. 5G). overexpression of miR-145 by mimics also reduced the AngII level in stretched cells, as shown in Fig 5H. Therefore, ACE was involved in stretch-induced miR-145 suppression likely via affecting AngII level in stretched cells.

## Discussion

We have made several important observations in the present study. First, 16% mechanical stretch reduced VSMCs contractile markers expression in cultured HASMCs. Secondly, miR-145 was implicated in stretch-induced VSMCs phenotype modulation by regulating the protein levels of myocardin and KLF4. Thirdly, and stretch-induced ERK1/2 activation and upregulation of ACE contributed to reduced miR-145 level by stretch.

Unlike some terminally differentiated cells, VSMCs retain plasticity and have the ability to modulate their phenotype in response to changes in the local environment. In response to vascular injury, VSMCs can switch their phenotype from a contractile to a synthetic state [2], [18]. Mechanical stretch due to blood pressure was also reported to modulate the VSMC phenotypic, but results from several studies were not consistent. The origin of cells, the ECM that embeds SMCs and the magnitude of stretch may explain the discrepancies. We found that 16% stretch significantly promoted cultured HASMC phenotypic modulation from a differentiated to dedifferentiated state as demonstrated by reduced expression of SMC-specific markers. Under normal conditions, VSMCs residing in the tunica media of human aorta undergo 9%∼12% stretch, mainly perform contractile functions and rarely have the ability to proliferate and migrate [19]. Here, application of 16% stretch to cultured HASMCs as occurs in hypertension in vivo revealed a synthetic phenotype. As well, ECM, in which VSMCs are embedded, also plays an important role in SMC differentiation. Under static conditions, the expression of contractile markers was increased with VSMC culture on laminin but not collagen or fibronectin [13], [20]. A previous report also revealed that VSMCs cultured on fibronectin or collagen, then subjected to stretch showed increased DNA synthesis; the opposite was true with culture on laminin [21]. In our study, we cultured HASMCs on collagen.

A large number of studies demonstrated that modulation of the VSMC phenotype plays a critical role in the pathogenesis of proliferative vascular disorders, including atherosclerosis and hypertension. However, the detailed mechanisms were not fully elucidated. Recently, several miRNAs were found involved in the regulation of cellular functions of ECs mediated by shear stress. The upregulation of miR-19a by shear stress contributed to inhibited EC proliferation [9], and its downregulation by pulsatile shear stress enhanced KLF2 expression and NO production [11]. Oscillatory shear stress increased miR-21 expression in ECs by activating AP-1, participating in the up-regulation of vascular cell adhesion molecule 1 and monocyte chemoattractant protein 1 [22]. As compared with roles of miRNAs with shear stress, those of stretch-mediated cellular functions of VSMCs are poorly understood. In our recent study, we found that elevated stretch increased miR-21 expression, implicated in the regulation of proliferation and apoptosis of cultured HASMCs [12]. From the effects of stretch on the VSMC phenotype and the identification of several miRNAs related to phenotype modulation of VSMCs, we hypothesized that miRNAs may also be involved in VSMC phenotypic switch mediated by stretch.

In the present study, among the many stretch-regulated miRNAs demonstrated by miRNA array, miR-21, -145, -31, -221, -24 were found involved in regulating the VSMC phenotype in special conditions [16], [23]–[25]. We further verified the changed expression of these miRNAs by qRT-PCR and confirmed that miR-21 was upregulated and miR-145 and -221 were downregulated by stretch. By gain- and loss-of-function approaches, we revealed that that expression of VSMC contractile markers was increased by miR-145 mimics in stretched cells but not affected by miR-21 inhibitor or miR-221 mimics. Thus, miR-145 may play a crucial role in stretch-induced HASMCs phenotype modulation. Indeed, miR-145 is the most powerful miRNA in regulating the VSMCs phenotype. The expression of VSMCs contractile markers such as SM-α-actin, CNN, and SM-major histocompatibility complex decreased with inhibition of miR-145 and increased with its overexpression, even under static conditions [26]. MiR-145 is cotranscribed with miR-143 from the same gene. Our data demonstrated that stretch increased the pri-miR-143/145 level as compared with static conditions, but the mature miR-143 level was not altered. Post-transcriptional regulating mechanisms may contribute to the effect of stretch on miR-143 expression.

SRF plays a critical role in regulating the expression of VSMC contractile marker genes by binding to the CArG box sequences in their promoter regions with its coactivators, including myocardin, ETS-like 1, MRTF-A and -B [27]–[30]. In our study, the expression of SRF and myocardin was decreased by 16% stretch. As well, SRF and myocardin were found to be positive regulators of miR-143/145 expression; therefore, inhibition of SRF and myocardin expression by stretch may contribute to decreased expression of VSMC contractile markers and miR-145 levels. Interesting, myocardin is also a target gene of miR-145 [31]; our data demonstrated that overexpression of miR-145 significantly enhanced the expression of VSMC contractile markers in stretched cells, and myocardin level was also increased, but SRF expression was not affected. Therefore, SRF was upstream of miR-145 expression, and other mechanisms could be involved in the regulation of SRF expression, but miR-145 could affect SRF activity by regulating myocardin level.

MiR-145 affects VSMC phenotypic modulation by targeting several transcriptional factors, including KLF4, KLF5 and myocardin. Therefore, miR-145 can self-regulate its expression by a feedback mechanism. KLF4 plays an important role in suppressing the expression of VSMC contractile markers by interacting with SRF and repressing myocardin expression [32], [33]. KLF4 expression could be induced by PDGF-BB, but the effect of stretch on its expression is not clear. In our study, we found that stretch increased KLF4 protein but not mRNA level, so regulation of KLF4 expression by stretch mainly occurred at the post-transcriptional level. overexpression of miR-145 induced KLF4 protein level in stretched cells, so stretch-inhibited miR-145 expression contributed to the increased KLF4 protein level. As well, the expression of VSMC contractile markers was increased in stretched cells with siRNA knockdown of KLF4 protein level, which demonstrates that KLF4 plays a role in the stretch-altered VSMC phenotype. As for other miR-145 targets, such as KLF5, another important repressor of myocardin may also play a role in stretch-altered VSMC phenotype but needs further investigation.

Upon stretch stimuli, several signaling pathways are activated to drive patterns of gene expression, thereby regulating cellular functions of VSMCs. ERK1/2, p38 MAPK and Akt pathways are activated rapidly by stretch as early as several minutes after treatment [34], [35], and these pathways were reported to be associated with VSMC differentiation. ERK1/2 activation induced by sustained stretch contributed to increased VSMC proliferation and migration [36]. The p38 MAPK pathway is involved in modified VSMC phenotype with epidermal growth factor treatment [37], and activated Akt by insulin-like growth factor can phosphorylate FOXO4, inducing its translocation from the nucleus to cytoplasm, thus promoting the expression of VSMC contractile markers [38]. However, the effects of these pathways on stretch-altered VSMC phenotype were not clear. In our study, we used specific Pharmacological inhibitors to suppress the activation of these pathways, results indicated only PD98059 significantly attenuated the stretch-suppressed expression of VSMC contractile markers and miR-145 level was also partially recovered. Therefore, the ERK1/2 pathway contributes at least in part to the inhibition of miR-145 expression by stretch.

Ang II is a potent stimuli for the proliferation of VSMCs and an important regulator of the phenotype modulation of VSMCs. As well, it is involved in regulating the activation of MAPK pathway in cultures VSMCs by stretch. Ang II is mainly generated by ACE converting Ang I to Ang II. Previous studies showed that pulsatile shear stress suppressed ACE expression and activity in cultured ECs [39], [40]; however, the effect of stretch on its expression is unclear. We found that 16% stretch enhanced ACE expression at the mRNA and protein levels. A recent study reported that the up-regulation of miR-145 was implicated in shear stress-reduced ACE expression [41]. Besides, Boettger et al have identified ACE as a major target of miR-143/145 [6]. In our study, overexpression of miR-145 by mimics also significantly attenuated stretch-induced up-regulation of ACE protein level but did not affect the mRNA level. Thus, inhibition of miR-145 contributes in part to stretch-induced upregulation of ACE expression, but other mechanisms may also play an important role, especially at the transcriptional level. These results indicated ACE was a mechanically sensitive gene and miR-145 was an important regulator of mechanical force mediated ACE expression. Ang II plays a role in stretch-induced ERK1/2 activation [42], and inhibition of Ang II generation by an ACE inhibitor or siRNA has obviously cardiovascular protective roles, including maintaining VSMC differentiation. We found that down-regulation of ACE expression by siRNA in stretched cells attenuated the stretch-suppressed miR-145 level, with increased expression of VSMC contractile markers. In addition, the increased AngII level in stretched cells was reduced by ACE siRNA or overexpression of miR-145. Therefore, stretch-suppressed miR-145 level was at least partially ACE dependent, there was likely presence of a negative feed-back loop that enables the inhibition of miR-145 expression, thus partially contributing to a phenotype switch from a differentiation to de-differentiation state of VSMC mediated by stretch, as shown in fig.5I. However, the detailed mechanisms of stretch-reduced miR-145 expression and the interaction between miR-145 and ACE need further investigation.

In conclusion, our data reveal a novel mechanism underlying the stretch-altered VSMC phenotype with injury. overexpression of miR-145 in vascular vessels exposed to elevated stretch as occurs in hypertension may represent a potential therapy to inhibit pathological vascular remodeling.
